# Regulation of Feeding and Metabolism by Neuropeptide F and Short Neuropeptide F in Invertebrates

**DOI:** 10.3389/fendo.2019.00064

**Published:** 2019-02-19

**Authors:** Melissa Fadda, Ilayda Hasakiogullari, Liesbet Temmerman, Isabel Beets, Sven Zels, Liliane Schoofs

**Affiliations:** Department of Biology, Functional Genomics and Proteomics, KU Leuven, Leuven, Belgium

**Keywords:** neuropeptide F, neuropeptide Y, short neuropeptide F, feeding behavior, neuropeptide evolution, G protein coupled receptor, protostomes, neuromodulation

## Abstract

Numerous neuropeptide systems have been implicated to coordinately control energy homeostasis, both centrally and peripherally. However, the vertebrate neuropeptide Y (NPY) system has emerged as the best described one regarding this biological process. The protostomian ortholog of NPY is neuropeptide F, characterized by an RXRF(Y)amide carboxyterminal motif. A second neuropeptide system is short NPF, characterized by an M/T/L/FRF(W)amide carboxyterminal motif. Although both short and long NPF neuropeptide systems display carboxyterminal sequence similarities, they are evolutionary distant and likely already arose as separate signaling systems in the common ancestor of deuterostomes and protostomes, indicating the functional importance of both. Both NPF and short-NPF systems seem to have roles in the coordination of feeding across bilaterian species, but during chordate evolution, the short NPF system appears to have been lost or evolved into the prolactin releasing peptide signaling system, which regulates feeding and has been suggested to be orthologous to sNPF. Here we review the roles of both NPF and sNPF systems in the regulation of feeding and metabolism in invertebrates.

## Introduction

The nomenclature of the two distinct neuropeptide families, short neuropeptide F (sNPF) on the one hand, and neuropeptide F (NPF) on the other hand, has led to confusing data and conclusions in literature. Whereas, NPF shares a common bilaterian ancestor with vertebrate neuropeptide Y (NPY), the sNPF system appears to be protostomian-specific according to (1). In this review, we summarize work on the regulation of feeding and metabolism by NPF and sNPF in invertebrates and attempt to clarify annotation ambiguities that have become apparent upon large-scale phylogenomic analyses of NPF and sNPF systems across bilaterians (1, 2).

### The NPY Family in Vertebrates

NPY is part of a large neuropeptide family that besides NPY, includes peptide YY (PYY) and the pancreatic polypeptide (PP) (3). The 36 amino acid NPY was isolated in 1982 from pig brain extracts (4) and was found to be widely distributed in the CNS of vertebrates (5, 6). Whereas, NPY is found at all levels of the brain-gut axis, PYY, and PP seem to be predominantly expressed by endocrine cells of the digestive system (7). The three peptides share a characteristic secondary structure called the pancreatic polypeptide-fold (PP-fold) (8) that is fundamental for the full activation of the NPY G protein coupled receptors (NPYRs). These GPCRs comprise the Y1, Y2, Y4, Y5, and Y6 subtypes (9, 10). The Y1, Y2, and Y5 receptor isoforms preferentially bind NPY and PYY (11), while Y4 is specific for PP (12). The Y6 receptor exists as a truncated inactive protein in most mammals, and is only a functionally active receptor in mice and rabbits (13). While NPY centrally promotes feeding and reduces energy expenditure, PYY and PP mediate satiety. In addition, NPY family neuropeptides have various other functions that go beyond the regulation of feeding and appetite (14).

### Discovery of NPF and sNPF Neuropeptides in Invertebrates

The first invertebrate NPY-like peptide was discovered in 1991 in the tapeworm *Moniezia expansa* using a C-terminally directed pancreatic polypeptide (PP) antiserum (15). HPLC purification of the PP-immunoreactive (IR) extract followed by automated Edman degradation sequencing identified a 39 amino acid tapeworm peptide that displays sequence similarities with the 36 amino acid vertebrate NPY (15). Based on its C-terminal sequence that ends with a phenylalanine (F) instead of a tyrosine (Y), this peptide was named neuropeptide F instead of neuropeptide Y. After this discovery, NPF-like peptides have been identified in other flatworms and in molluscs (16–20), all of which typically display an RPRF-amide C-terminal sequence and a length ranging from 36 to 40 amino acids.

The first sNPF peptides were discovered in insects, including the Colorado potato beetle *Leptinotarsa decemlineata* and the desert locust *Schistocerca gregaria* (21, 22) using antisera raised against *M. expansa* “long” NPF (23). These insect peptides consist of only 8 to 10 amino acids instead of 36 to 40 amino acids as typical for vertebrate NPY and flatworm or mollusc NPF. Based on their carboxyterminal RLRFamide sequence, which is similar to the RPRFamide motif of the “long” NPFs from flatworms and molluscs (15), they were designated “short” NPFs or sNPFs (24).

### Discovery of NPF and sNPF Receptors in Invertebrates

NPF receptors (NPFRs) were initially cloned from the brain of the pond snail *Lymnaea stagnalis* (25) and subsequently from *Drosophila* larvae (26, 27). Both receptors retained the typical features of vertebrate NPYRs and they showed the highest homology to the mammalian NPYR 2 isoform (28). Upon expression of these NPFRs in Chinese Hamster Ovary (CHO) cells, it was found that the respective NPF peptides inhibit forskolin-stimulated adenylyl cyclase activity, in accordance with vertebrate NPYRs signaling through G_i/o_ small proteins (25, 26, 29). In addition, the *Drosophila* NPF receptor can be activated by mammalian NPY-type neuropeptides when expressed in *Xenopus* oocytes (27).

The first sNPF receptor (sNPFR) was cloned from *D. melanogaster* (30) and later on from the fire ant *Solenopsis invicta* (31) and the mosquito *Anopheles gambiae* (32). Different *Drosophila* sNPF variants elicit a calcium response in CHO cells (30, 33) or in *Xenopus* oocytes (26, 34) when these are transformed to express the *Drosophila* sNPFR.

### Issues With Nomenclature

After the cloning of a long 36 amino acid NPF neuropeptide precursor in *Drosophila melanogaster* (35) and the sequencing of the *Drosophila* genome (36), it became evident that not all NPF/NPY-immunoreactive peptides that were designated as NPF were actually long NPFs such as those isolated from *M. expansa* and *D. melanogaster*. This led to the introduction of the terms long NPF (or simply NPF) and short NPF (sNPF) (24, 37). Alignment studies of neuropeptide precursors from both vertebrates and invertebrates pointed out the diversity in the consensus sequences for sNPF and NPF (38, 39), and suggested that short NPFs are restricted to protostomian phyla, while long NPFs are conserved across bilaterians.

In the past, several studies in insects have demonstrated overlapping functions of NPF and sNPF signaling with respect to feeding and metabolism, suggesting a common evolutionary origin of sNPF and NPF neuropeptides and receptors. This hypothesis was also supported by structural similarities between both invertebrate sNPF and NPF receptors and vertebrate NPY receptors (NPYR) (in particular with vertebrate NPY2R) (28, 39). Recent phylogenomic analyses of neuropeptide receptor families in bilaterians, however, show that sNPF and NFP receptors share only a distant common ancestor, explaining the structural similarities between both.

**Figure 1:**
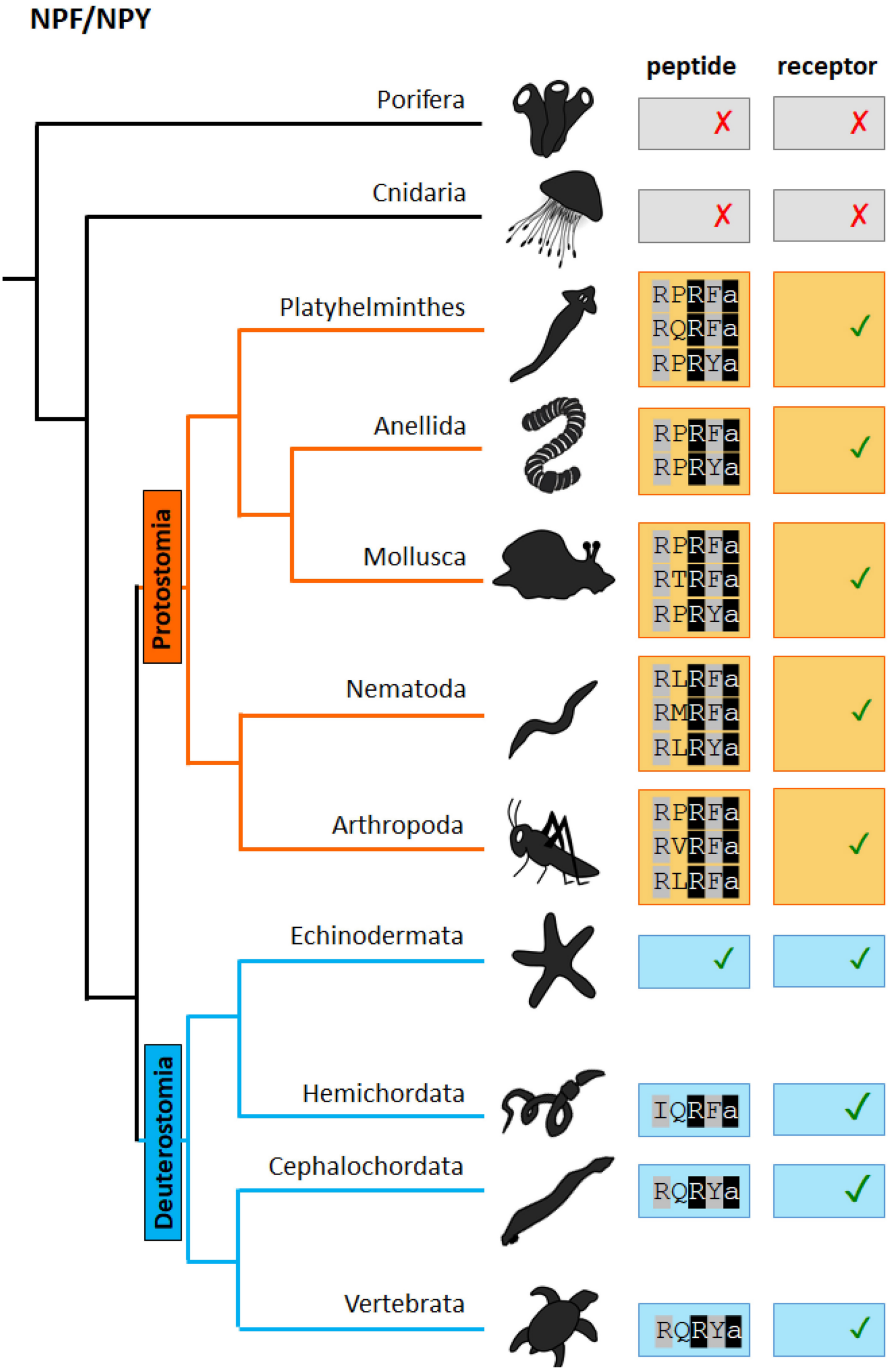
Scheme of the presence of NPF/NPY neuropeptides and receptors in distinct phyla of the metazoan evolutionary tree. The NPF(Y) C-terminal sequence motif is indicated. “✓” and “✗,” respectively represent the presence or absence of the peptide/receptor in the corresponding phylum. Evidence for the presence of both NPY/F peptides and receptors in cephalochordates and hemichordates can be found in Elphick et al. (40).

**Figure 2:**
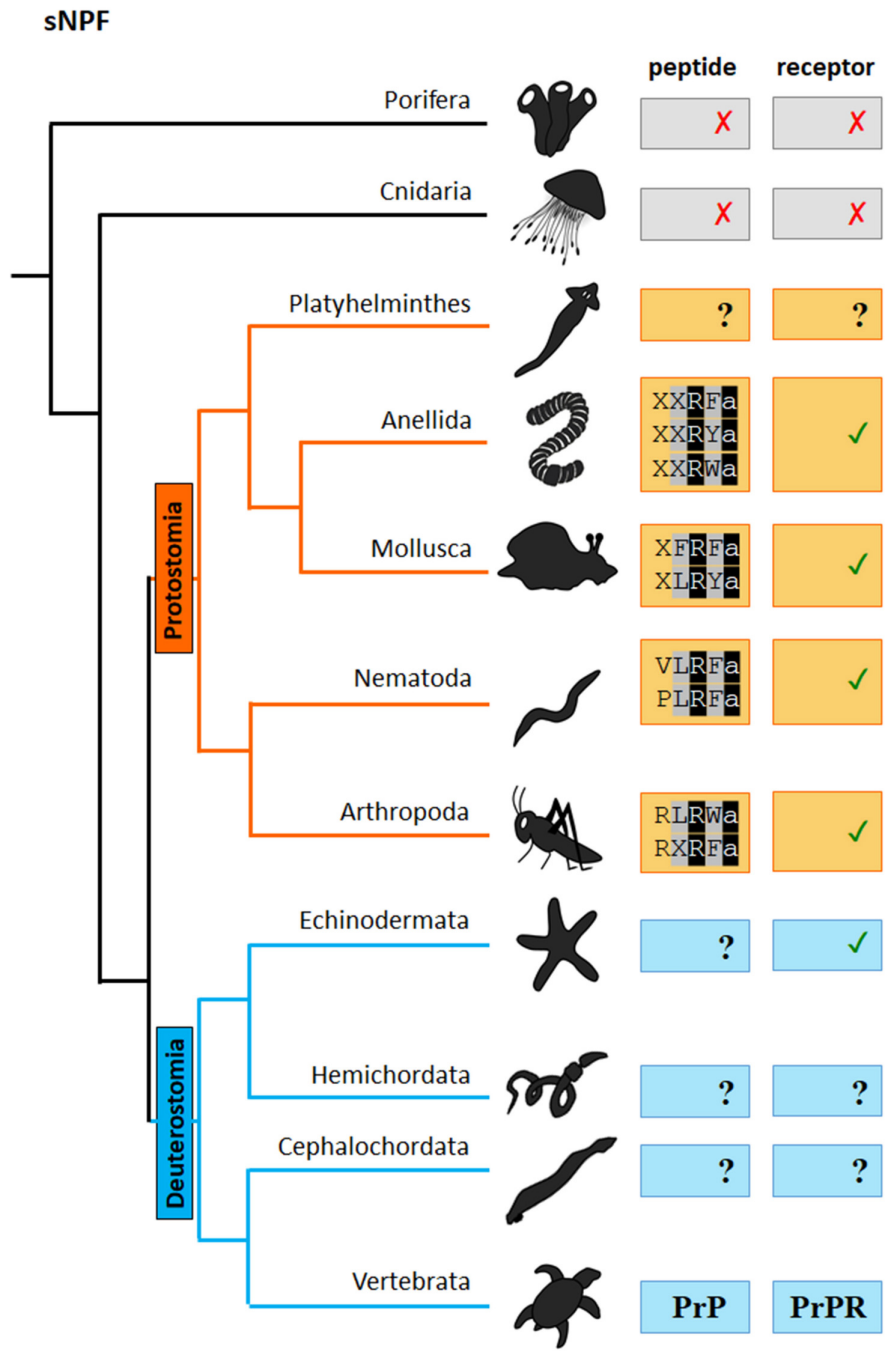
Scheme of the presence of sNPF neuropeptides and receptors in distinct phyla of the metazoan evolutionary tree. “✓” and “✗,” respectively represent the presence or absence of the peptide/receptor in the corresponding phylum. “**?**” indicates that the presence of the peptide/receptor is not clear or has so far not been investigated. PrP: prolactin. PrPR: prolactin receptor. The sNPF sequence motif is indicated. The first X in the Annelida peptide motif represents a hydrophobic amino acid, while the second X indicates a Phe, Leu or Met amino acid residue. The X in the Mollusca peptide motif represents a hydrophobic amino acid. The X in the Arthropoda peptide motif represents a Leu or Ile amino acid residue.

Both NPF and sNPF signaling systems in invertebrates have been implicated in the regulation of a diverse array of biological processes including reproduction, growth, nociception, circadian clock, learning, feeding and metabolism and they function mainly as neuromodulators or neurohormones [For an extensive review see (39)]. Here we will review past research on NPF and sNPF in the regulation of feeding-related behaviors and metabolism in protostomes. For each discussed phylum, we will briefly introduce the initial characterization of the sNPF and NPF signaling systems, followed by the current knowledge on their function(s) in feeding and metabolism. We will make a clear distinction between NPF and sNPF and will point to annotation ambiguities where appropriate. Clarifying annotation errors is not a goal here but will be crucial to further understand the evolution of the functions of sNPF and NPF neuropeptides signaling systems. Although the current picture is still not entirely clear due to insufficient genome information from all phyla, we now have increased insight in the evolutionary history of sNPF and NPF systems (Figures 1, 2). It is clear that NPF and sNPF are distinct neuropeptides systems, both involved in the regulation of feeding and metabolism. Both systems branched off from their common ancestor early in evolution, prior to the split of the deuterostome and protostome lineages. Therefore, NPF and sNPF signaling systems are a prime example to ask questions on the sustained evolutionary selection for both systems in protostomes in contrast to the deuterostomian chordate lineage where only the NPF orthologs (NPYs) have been conserved and even have been duplicated multiple times, but where the sNPF system seems to have been lost or evolved into the prolactin-releasing peptide system.

## The (long) NPF SIGNALING system and its Role in Feeding and Metabolism

**Figure 3:**
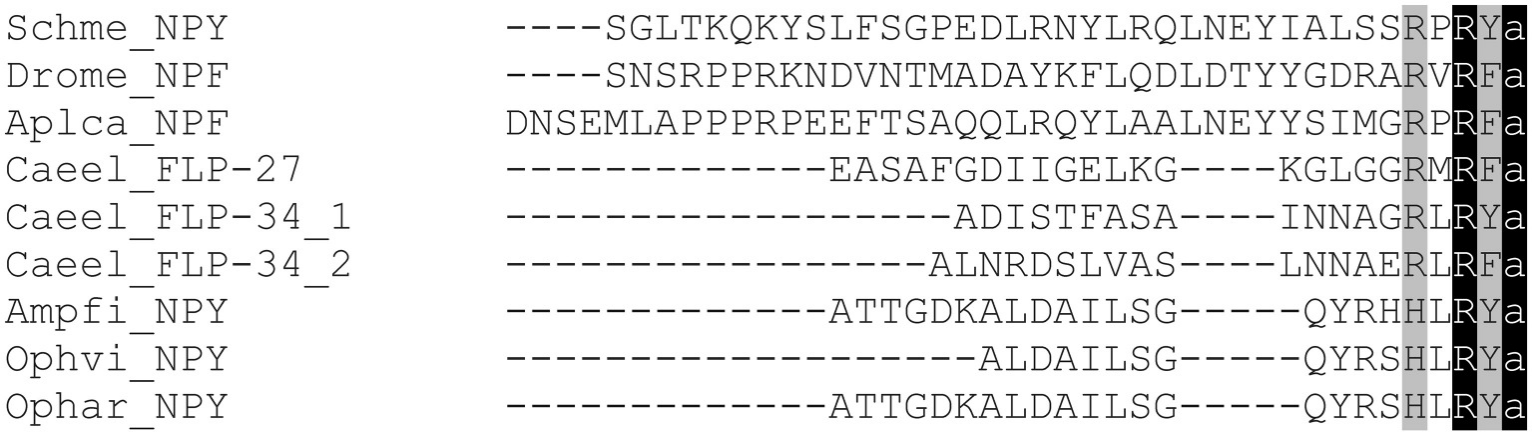
Amino acid sequence alignment of representative NPFs from different invertebrate phyla. Genus and species abbreviations used in the alignment are: Schme, *Schmidtea mediterranea;* Drome, *Drosophila melanogaster;* Aplca*, Aplysia californica;* Caeel, *Caenorhabditis elegans;* Ampfi, *Amphiura filiformis;* Ophvi, *Ophionotus victoriae;* Ophar, *Ophiopsila aranea*. Identical residues are highlighted in black and conserved residues in gray.

Figure 3 shows an alignment of representative NPF neuropeptides that have been biochemically isolated or identified by genome sequencing (for a broader overview of protostomian sNPF and NPF peptides, see Supplementary Tables 1, 2). NPFs generally consist of more than 28 amino acids, apart from the shorter predicted *C. elegans* orthologs and some truncated insect NPFs, and share the common RXRF/Yamide carboxyterminal motif (Figure 1).

### NPF Signaling in *D. melanogaster*

Most studies on the regulation of feeding and metabolism by NPF were conducted in the genetically tractable model organism, *D. melanogaster*. In 1992, cloning and functional expression of two distinct *D. melanogaster* NPY-like receptors revealed that they could be activated by mammalian NPY and peptide YY (27). The first insect NPY-like peptide was also identified in *D. melanogaster*, and consisted of 36 amino acids with a characteristic RVRFa carboxyterminal sequence (35). The C-terminal F residue, instead of the vertebrate NPY-defining Y residue, prompted the name conversion from NPY to NPF, previously adopted in other protostomes as well (15). In 2002, *Drosophila* NPF was shown to dose-dependently activate the *Drosophila* NPY-like receptor NPFR when expressed in CHO cells (26). By means of immunocytochemistry and *in situ* hybridization, NPF was localized in the midgut and brain of *D. melanogaster*, suggesting a role in feeding, digestion and/or metabolism (35).

The first experimental evidence for a role of *Drosophila* NPF in the regulation of metabolism was provided by analysis of *npf* transcription levels following sugar exposure. A sugar-rich diet fed to *D. melanogaster* larvae evoked *npf* expression in two distinct neurons of the suboesophageal ganglion. Additional experiments with mutant flies deficient in sugar sensing, highlighted that not sugar ingestion, but taste perception of sugar was essential for *npf* expression (41). Subsequent studies showed that *npf* expression is high in young, foraging larvae and low in older larvae that display food aversion and burrowing. Experimentally induced overexpression and downregulation of *npf* transcript levels shifted these stage-specific feeding-related phenotypes (42).

In-depth characterization of the NPF signaling pathway revealed that NPF functions downstream of insulin signaling to regulate feeding in *Drosophila* larvae. NPF does not specifically influence total food intake, but may rather regulate food choice behavior (43, 44). NPF neurons are hypothesized to modulate the reward circuit to acquire lower-quality foods upon food deprivation (43). NPF signaling through its NPFR receptor promotes the intake of noxious food in starved flies and inhibits the aversive response that is normally elicited. In satiated flies, however, activation of the insulin signaling pathway results in the inhibition of the NPF-induced feeding response toward noxious food (44). NPF thus regulates a feeding response, that integrates both food attractiveness and hunger state (45). Together, these studies show that lower quality food or noxious food can evoke an NPF-mediated feeding response in starved flies, while in metabolically satiated flies, NPF is inhibited resulting in the intake of higher quality food (44, 45).

NPF signaling is also required at the intersection of feeding and stress, namely for the regulation of cold-resistant feeding behavior (46). NPFR1 is expressed in fructose-responsive sensory neurons in the thorax, suggesting that NPF may modulate these neurons directly (47). NPF could modulate the activity of the transient receptor potential ion channel A (TRPA) called PAIN and inhibits the regular avoidance response to aversive stimuli.

NPFR1 colocalizes with the majority of dopaminergic neurons in the larval *D. melanogaster* CNS, suggesting extensive interplay between these two signaling pathways (48, 49). Firstly, NPF expression in the brain of *D. melanogaster* may represent the food-deprived state, in which dopaminergic neurons in the mushroom bodies (MBs) promote appetitive memory formation (48). Secondly, NPF signaling modulates dopaminergic transmission in *D. melanogaster* in appetitive olfaction. NPFR1-expressing dopaminergic neurons display projections toward DL2-lateral horn (LH) neurons that receive olfactory inputs, and modulation of these neurons by NPF is instrumental to stimulate odor-induced appetitive feeding (49). NPF thus can stimulate (MBs) or inhibit (DL2-LH neurons) dopaminergic signaling in functionally distinct neurons even if it does not appear to be implicated in octopaminergic regulation of feeding motivation (50). In relation to this, NPF has recently been proposed to modulate odor-aroused appetitive behavior through a newly characterized DA/NPF-mediated circuit (51). When an appetitive odorant is perceived, the information is integrated in the DL2 neurons and transmitted to Dop1R1 neurons that express NPF. The dorsomedial pair of NPF neurons are essential for the proper manifestation of odor-aroused appetitive behavior (51). NPF signaling also seems to be implicated in odorant detection and is required for sensitization and correct odorant perception by a subset of olfactory neurons named antennal basiconics (ab)3A that besides NPFR also express the olfactory receptor (OR)22a, which responds to different fruity odorants (52). Related to motivation, sucralose can cause stimulation of a gustatory receptor that signals via dopamine and octopamine to activate a reward pathway in which NPF is also implicated. Supplementation of sucralose to the fly's diet caused an imbalance between energy and food sweetness that is signaled by the sweet taste receptor *Gr64a*. A mechanistic analysis of this sucralose response identified the NPF system as a critical downstream component in this pathway, confirming a conserved role for NPF in sucralose-sweetened food intake stimulation (53).

Since NPF is involved in the regulation of feeding and metabolism on multiple levels in *D. melanogaster*, its effect on obesity has also been studied. Leptin is a neuropeptide involved in the regulation of food attraction, food intake and body weight and its *Drosophila* homolog is Upd-1. Interestingly, the Upd-1 receptor *Domeless* is expressed in the brain's NPF-neurons. The odor-activated food response that is normally elicited by NPF and regulated by internal metabolic status (45), is perturbed in flies lacking Upd-1 in the NPF neurons. When the upstream regulation by *Upd-1* and *Domeless* is absent, NPF-neurons do not register the satiety status of the flies and always display odor responses on the same level as starved flies, leading to overconsumption of food and obesity (54). The enzymatic cofactor tetrahydrobiopterin (BH4) is another compound that can affect the activity of NPF neurons in satiety. BH4, synthesized by the adipose tissue, inhibits NPF signaling by blocking its release and thus induces satiety (55).

In *Drosophila*, NPF modulates both food intake and wakefulness (56). In particular, NPF signaling plays a fundamental role in wake extension during deprived feeding conditions, in order to facilitate the search of new food sources. However, the neuron clusters for wake and feeding phenotypes were completely unrelated, suggesting an independent regulation of the two behaviors (56).

### NPF Signaling in Other Insects

Confusion between NPF and sNPF partly results from insect research where a clear distinction between both neuropeptide systems remained difficult for a long time, due to the identification of so-called “NPY-like” neuropeptides that differed in length from vertebrate NPY. Studies in the insects, *Locusta migratoria, Schistocerca gregaria* (57), and *Helicoverpa zea* (58), in which only a C-terminal fragment of the “full-length” NPF was isolated and identified by mass spectrometry, contributed to this confusion. Identification of the respective neuropeptide precursor sequences by RNAseq and genome sequencing has clarified this issue. Comparison of the coding sequences of these peptides shows that they are truncated forms of larger NPF neuropeptides and that the shorter C-terminal fragments may thus either be artifacts from the extraction procedure or may be created by extensive posttranslational processing *in vivo* (57, 58).

The earliest indications of NPF being involved in feeding and metabolic regulation in various insect species were based on immunocytochemical localization studies. Its expression in the digestive system or its temporal regulation of expression in response to food intake suggested a regulatory role of NPF in the control of feeding and metabolism. In the yellow fever mosquito, *Aedes aegypti*, NPF-IR was detected in the midgut and in the suboesophageal ganglion where the regulation of food intake resides. Analysis and sequencing of the immunoreactive peptides from head and midgut extracts confirmed the presence of NPF. *A. aegypti* NPF inhibits transepithelial ion transport in the anterior stomach, suggesting a function in the digestive system (59). The titer of *A. aegypti* NPF in the haemolymph is influenced by feeding and decreases drastically after a blood meal (60). Recent studies indicate that genetic and pharmacological disruption of the mosquito NPF pathway results in abnormal host-seeking behavior and blood-feeding (61).

In *S. gregaria*, however, injection of NPF increases food intake, while RNA interference of *npf* transcripts decreases food intake. These treatments resulted in weight gain for the peptide-injected group and stunted weight gain for the knockdown group, which implies a stimulatory role of NPF in feeding (62). *S. gregaria* NPF transcription is spatiotemporally regulated in response to feeding, with high levels in starved animals that drop in the brain, optic lobes, and midgut upon feeding, but significantly rise in the suboesophageal ganglion (62). Also in *Bombyx mori*, knockdown of NPFR resulted in a reduction of food intake and growth, pointing toward a role for NPF as a positive regulator of feeding (63).

A typical long NPF as well as its truncated form have been found in *Rhodnius prolixus* (64). NPF-like-IR is present in the stomatogastric nervous system of *Rhodnius*, which regulates feeding. In addition, radioimmunoassay quantification showed a decrease in the intensity of NPF-like IR material in the cell bodies and axons after feeding, suggesting a release into the haemolymph (65).

In the honey bee *Apis mellifera* (66) and in the wasp *Nasonia vitripennis* (67), the NPF sequence does not retain the canonical RPRF/Yamide C-terminal sequence, displaying instead a C-terminal KARYamide motif. Hymenopteran NPF protein precursor sequences show nevertheless clear similarities with NPF/Y precursors of other invertebrate phyla, having YY residues in position 30–31 as well as other conserved amino acids. Interestingly, genomes of hymenopteran species do not seem to encode a clear ortholog of the NPF receptor. It is therefore not unthinkable that evolutionary pressure drastically shaped the so far unknown receptor in hymenopterans into a GPCR with unrecognizable orthology and that receptor-neuropeptide co-evolution resulted in a modification of its ligand.

In *A. mellifera*, NPF levels differ according to the age and tasks of the worker bees. Younger workers providing brood care display low NPF intensities in *in situ* hybridization and qPCR analysis, while older workers that are responsible for foraging display higher expression levels (68). This may suggest a stimulatory role for NPF in food searching and foraging behavior of worker bees, but experiments providing a causal relationship are currently lacking.

### NPF Signaling in Other Arthropods

Information about NPF orthologs in arthropods outside the insect orders is currently increasing. Bioinformatic analysis of genomes and transcriptomes revealed the presence of NPF and NPF receptors in chelicerates, including spiders, scorpions and mites (69). An *in silico* analysis using the *D. melanogaster npf* transcript as a query against the crustacean expressed sequence tags (ESTs) database revealed putative hits for the shrimp *Marsupenaeus japonicus* and the water flea *Daphnia magna* (70). The predicted pro-peptides, respectively, encode a 32 and 38 amino acid NPF peptide, which both display the typical RPRFamide carboxy terminus. A similar analysis also revealed an NPF ortholog in *Daphnia pulex* (71). In another study, *npf* transcripts have been isolated from mixed eyestalk ganglia of *Litopenaeus vannamei* and from the brains of *Melicertus marginatus* (72). Both penaeid shrimp species have two identical transcript sequences and one transcript differs from the other by an in-frame 37-amino acid insertion in the middle of the coding sequence of NPF. Both transcripts are broadly distributed in the nervous system of the animals and the short one is also expressed in some of the midgut samples. A diet supplemented with the shorter NPF induced a significant increase in food intake and growth in juvenile *L. vannamei* suggesting an orexigenic action for the NPF (72).

While a neuropeptide from the crab *Pugettia productav* has been suggested to resemble vertebrate NPY because of its SQRYamide carboxyterminal sequence (73), recent phylogenetic analysis indicates that the RYamide neuropeptide family, which is widespread in arthropods (74, 75), is evolutionarily distinct from the NPF/NPY peptide family (1).

### NPF Signaling in Nematodes

In nematodes, neuropeptide signaling systems suggested to be orthologous to NPY/NPF were initially discovered in *C. elegans* (76, 77). Neuropeptide receptor 1 (NPR-1) was cloned from a solitary wild-type strain and firstly assigned to the NPYR family based on its sequence similarity with the vertebrate NPYR groups (78). In a following whole-genome analysis study, using the *L. stagnalis* lymnokinin GPCR as a query, *C. elegans* NPR-1, NPR-2, NPR-3, NPR-4, NPR-5, NPR-6, NPR-7, NPR-8, NPR-10, NPR-11, NPR-12, and NPR-13 were all annotated as NPY-like receptors (76, 79). Two of these NPRs, NPR-11, and NPR-12 cluster closest with the *Drosophila* NPFR as shown by phylogenetic tree analysis (76) and thus appear to be true NPF homologs in *C. elegans*. These NPFR orthologs are also encoded in the genomes of all other nematodes investigated (76, 80).

A specific role in feeding or metabolism has not been investigated for the nematode NPF system. The only functional study that has been carried out is on the NPFR ortholog, NPR-11, which is involved in local search behavior of *C. elegans* when the animal is removed from its bacterial food source (81). In this work, the neuropeptide-like protein 1 (NLP-1), although not containing a peptide with a typical NPF-motif, was reported to act upon NPR-11 in the AIA interneuron to modulate local search behavior.

### NPF Signaling in Platyhelminths

The first evidence for NPY orthologs in platyhelminths was found in the central and peripheral nervous system of the cestode *M. expansa*, from which a 39 amino acid peptide was identified by plasma desorption mass spectrometry (PDMS) upon isolation monitored by pancreatic polypeptide (PP) antiserum (15). The primary structure of this *M. expansa* NPF neuropeptide displays strong sequence similarities with vertebrate neuropeptide Y (15). Subsequently, immunochemical analysis using antibodies against *M. expansa* NPF and against vertebrate PP, Peptide tyrosine tyrosine (PYY) and Substance P (SP) revealed the presence of immunoreactive substances in other cestodes (82–85) and other flatworm classes, including turbellarians (16, 86, 87), monogeneans (88–91), and trematodes (17, 92–94). So far, the immunopositive material has only been identified in *Arthurdendyus triangulates* (16), *Schistosoma mansoni* and in *Schistosoma japonicum* (17). All three platyhelminth peptides have a length between 36 and 39 amino acids and display the NPF-typical RPRF carboxyterminal sequence. In addition, phylogenetic tree analysis identified several GPCRs similar to NPF/Y receptors in the *S. mansoni* GPCR repertoire (95).

Studies on a putative role of NPF in feeding and metabolism have as yet not been performed in platyhelminths, but PP-, PYY-, and *M. expansa* NPF-IR is present in neurons innervating the oral and ventral suckers of *Schistosoma mansoni* (94), in the pharynx musculature of *Procerodes littoralis* (87) and in fibers and cells of the intestinal wall of *Microstomum lineare* (86), suggesting a possible role in the modulation of food intake and digestion. However, these results should be interpreted with caution as cross immunoreactivity with other neuropeptides cannot be excluded.

### NPF Signaling in Molluscs

One year after the identification of NPF in platyhelminths, several independent studies demonstrated the presence of NPF-like peptides in different species of molluscs. Following the detection of PP-IR in circumoesophageal ganglia extracts of the garden snail *Helix aspersa* (18), PMDS and automated Edman degradation led to the identification of an of 39 amino acid peptide that displays pronounced sequence similarities with NPF (18). Two additional NPF orthologs were discovered in the sea slug *Aplysia californica* (19) and in the cephalopod *Loligo vulgaris* (20). All display the C-terminal RPRF motif that typifies invertebrate NPF (19). Interestingly, gel permeation chromatography of the squid extract resolved two peaks of PP-IR with different molecular weights. One was suggested to contain (long) NPF and the second one contained a nine amino acid peptide harboring an N-terminal tyrosine and the NPF-typical RPRF C-terminus, but lacking the PP-fold structure (PYF) (20). This short peptide is either the result of an extraction artifact or the product from another as yet unknown neuropeptide precursor.

The only NPY/NPF receptor homolog known in molluscs to date has been cloned in the snail *L. stagnalis* (25). It is broadly expressed in the CNS. The 39-amino acid *L. stagnalis* NPF, which displays the typical RPRF C-terminal sequence and the N-terminal proline functionally activates this receptor, when expressed Chinese Hamster Ovary (CHO) cells (25).

A role for NPF in feeding and metabolism has been reported in various molluscan species. Injection of *A. californica* NPY (Aplca-NPY or better Aplca-NPF) into the hemocoel of the animal resulted in a dose-dependent reduction of food intake (96). In *A. californica*, the feeding process consists of an initial ingestion program triggered by the presence of food and initiated by the cerebral ganglion that is progressively converted to an egestion program when the esophagus and the gut signal satiation. The change in feeding state depends on the activity of the feeding central pattern generator (CPG) that receives signals derived from cerebral and buccal ganglia. In particular, the cerebral-buccal interneuron 2, located in the cerebral ganglion, initiates the ingestion program promoting the activity of the CPG interneuron B40. When the animal approaches satiation, the release of the Aplca-NPF enhances the egestion program by reducing the activity of B40 and promoting the activation of the egestion-promoting neuron B20, also located in the CPG. Aplca-NPF thus acts as a satiety signal that balances the activity of the antagonistic CPG interneurons B40 and B20 to potentiate the egestion program (96). Although the role of NPY as an orexigenic agent has been widely demonstrated in vertebrates (97–99) and in *D. melanogaster* (35, 41), the *A. californica* ortholog seems to display the opposite function. In humans and rodents, the gut released peptide YY_3−36_ isoform (PYY_3−36_) has been reported to inhibit food intake (100). It is produced postprandially by the intestinal L-cells in proportion to the calories ingested and, upon release in the blood circulation, it reaches the arcuate nucleus of the hypothalamus (101). Here, PYY_3−36_ acts on NPY2R, which is an inhibitory presynaptic receptor expressed in NPY neurons, modulating the activity of the NPY orexigenic pathway and leading to decrease in appetite (100, 101). Therefore, the localization and the functional activity of Aplca-NPF seems to be more closely related to the vertebrate PYY_3−36_ than to NPY (96). However, administration of *L. stagnalis* NPY (Lymst-NPY or better Lymst-NPF) in the snail led to a reduction in growth and reproduction without clear short-term effects on food intake (102). Thus, NPF regulation of energy flows appears to be conserved in *L. stagnalis*, while the regulation of food intake seems to be controlled by a leptin-like factor named *L. stagnalis* storage feedback factor (Lymst-SFF). This peptide is released from the glycogen cells of the mantle edge lining the shell of the animal that represents the only energy reserve in *L. stagnalis*.

A recent study on filter feeding has reported a “vertebrate-like” action of *Ruditapes philippinarum* NPF (Rudph-NPF) (103). Bivalves actively control food uptake by adjusting the filtration rate according to internal metabolic signaling. In accordance with this, injection of Rudph-NPF in the hemocoel resulted in a dose-dependent increase of 23% of filtration rate. In addition, a qPCR analysis demonstrated a large increase of Rudph-NPF mRNA levels in the visceral ganglion during starvation that rapidly declined after feeding, resembling the NPY expression pattern in vertebrates (104–107). Rudph-NPF injection was also associated with an increase in insulin and monoamine (serotonin and dopamine) levels, suggesting a coordinated regulation of filter feeding by multiple internal signaling pathways. In conclusion, a controversial role of mollusc NPF in the control of feeding has emerged from the works reviewed here: Aplca-NPF has an anorexigenic effect and seems to be functionally related to the vertebrate PYY_3−36_ isoform, while the Rudph-NPF showed an orexigenic effect more related to the vertebrate NPY. More interestingly, the Lymst-NPF does not exhibit any clear regulation of food intake, but modulates growth and reproduction. A possible explanation for this diversity could be related to the differential tissue expression of NPF receptors that are present in the different mollusc species. Since only the NPF receptor of *L. stagnalis* has been characterized so far, additional receptor identification studies are needed to elucidate the role of NPF in the regulation of feeding in molluscs.

### NPF Signaling in Annelids

The presence of NPF(Y) orthologs in annelids has been assessed by bioinformatics in the genomes of the earthworm *Lumbricus rubellus*, the polychaete worms *Alvinella pompejana* and *Capitella telata*, and the leech *Helobdella robusta*as (108, 109). In all species the predicted NPFs range from 29 to 43 amino acids in length and in the oligo-and polychaetes they display the NPF-typical RPRFamide carboxy terminus. In addition, a large transcriptome analysis of *Platynereis dumerilii* identified four NPF paralogous named NPY-1 to 4 of 38 to 48 amino acids in length (110). All the four genes show the typical RPRFamide carboxyterminal sequence and a partial sequence of the NPY-4 pro-neuropeptides has been confirmed by mass spectrometry (110). Although the NPY-4 receptor 1 does not seem to cluster with vertebrate NPY receptors, screening of *P. dumerilii* orphan GPCRs against *Platynereis* peptide mixtures revealed that this receptor is activated by three of NPF paralogues (NPY-1, NPY-3 and NPY-4, all displaying the NPF-typical RPRFamide carboxyterminal motif) in a calcium-based cellular receptor assay (111).

NPF encoding genes seem to have expanded in annelids. In *Capitella*, three predicted paralogous genes encode NPF (109), and in *Helobdella five* paralogues have been predicted, of which one is probably a pseudogene. Interestingly, three of these five paralogous sequences contain an NPY-typical RPRYamide C-terminal motif instead of the canonical invertebrate RPRFamide sequence (109). It has been shown that leech salivary gland neuropeptides, including NPY, aid in suppressing inflammation in their hosts from which they suck blood (112) and therefore, some of the diversified NPF(Y)s in leeches might be the result of convergent evolution because of their ectoparasitic lifestyle. An alternative explanation resides in the genome organization, gene structure, and functional content of the Spiralia (annelids and molluscs), which appear to be more similar to those of some invertebrate deuterostome genomes (*Amphioxus* and sea urchin) as well as non-bilaterian metazoans (such as cnidarians, sponges, and placozoans) than to those of ecdysozoan an platyhelminth protostomes that have been sequenced to date (113, 114). On the other hand, similarity-based clustering for neuropeptides and GPCRs of metazoans revealed that the predicted NPF peptides and receptors of annelids strongly cluster with the ones of arthropods, platyhelminths, and molluscs (2). Evidently, for a comprehensive genomic understanding of the metazoan radiation, a far larger sampling of genomes will be needed. Since only bioinformatic and phylogenetic analyses have been performed on annelids NPFs, their function remains elusive as yet.

### NPF Signaling in Echinoderms

One of the first investigations on the presence of evolutionarily conserved neuropeptides in echinoderms has been carried out in the starfish *Marthasterias glacialis* (115). Among the tested antisera, porcine PYY and human PP-like IR was found to be present in the endocrine cells and the basoepithelial plexus of the digestive tract. Analysis of the sea urchin *Strongylocentrotus purpuratus* genome revealed the existence of NPF(Y) receptors (116, 117). In ambulacrarians that comprise both echinoderms and hemichordates both NPY receptors and NPY neuropeptides (in *Saccoglossus kowalevskii*) have been predicted (2) (1, 118). In addition, a bioinformatic study focussing on echinoderms has recently shown the presence of NPY orthologs in *Ophiopsila aranea, Asterias rubens* and *Amphiura filiformis* (119). The aligned peptides lack the RQRYamide canonical consensus sequence of vertebrate NPYs but do contain a conserved RYamide carboxy terminus as well as other key amino acids (Figure 3). Functional studies on echinoderm NPY are currently lacking.

## The short NPF Signaling System and its Role in Feeding and Metabolism

**Figure 4:**
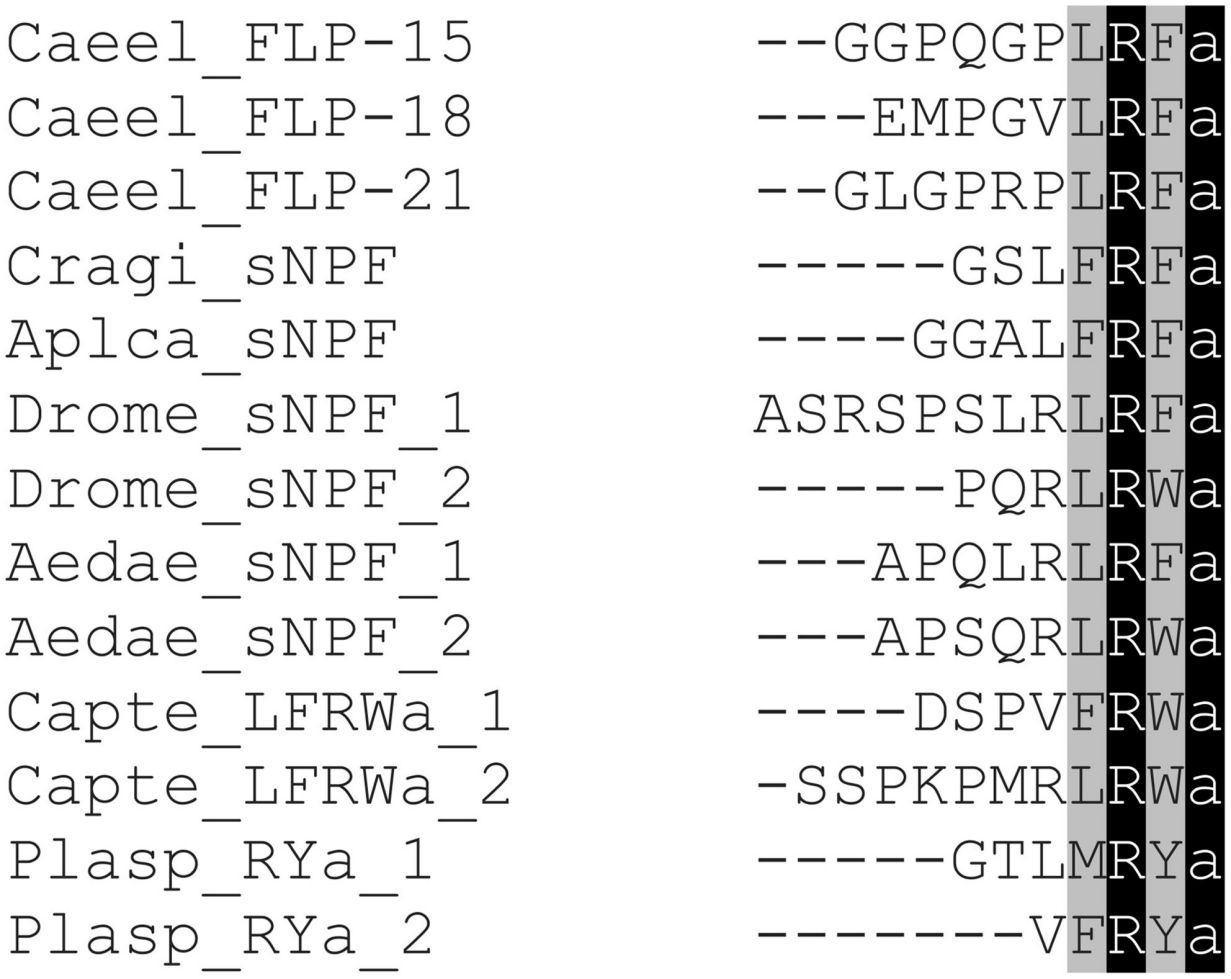
Amino acid sequence alignment of representatives of sNPFs from different invertebrate phyla. Genus and species abbreviations used in the alignment are: Caeel, *Caenorhabditis elegans;* Cragi, *Crassostrea gigas;* Aplca, *Aplysia californica;* Drome, *Drosophila melanogaster;* Aedae, *Aedes aegypti;* Capte, *Capitella telata;* Plasp, *Platynereis* species. Identical residues are highlighted in black and conserved residues in gray.

Short NPFs are short neuropeptides of 8 to12 amino acids in length and display the typical C-terminal consensus sequence M/T/L/FRFa (Figures 2, 4). As mentioned above short NPF and long NPF (NPY) are evolutionarily distant from one another. It has long been assumed that sNPF neuropeptides are confined to arthropods (http://www.neurostresspep.eu), but it has now become clear that both sNPF and NPF systems already originated in the common ancestor of protostomes and deuterostomes. Whereas, the long NPF (NPY) signaling system has been retained in both lineages, the short NPF signaling system appears to have been highly conserved across protostomes, and possibly in echinoderms (deuterostomes) as well. This raises important questions as to whether the regulatory functions of sNPF and NPF systems can be correlated with the differential lifestyles and environments of respective species. Although the sNPF system seems to have been lost in vertebrates, sNPF receptors have been shown to cluster with vertebrate prolactin-releasing peptide receptors (2), which also have a prominent role in the regulation of feeding behavior. Further research is needed to clarify this issue.

### sNPF Signaling in *D. melanogaster*

In *Drosophila*, four sNPF neuropeptides were predicted from the genome sequence (120), and later on identified by mass spectrometry-based peptidomics (121). Their cognate receptor was identified by means of a calcium-based receptor assay in CHO cells (30).

The use of *snpf* mutant flies showed that sNPF is involved in the regulation of food intake and body size in *D. melanogaster*. sNPF increases food intake in larval and adult flies, yet does not prolong the feeding period in larvae or modulate food preferences, as opposed to NPF (122). Pathway analysis of the sNPF signaling system revealed an interaction with insulin signaling regulating growth. sNPF activates extracellular-activated receptor kinases (ERKs) in insulin-producing cells (IPCs), which in turn modulate the expression of insulin (123, 124). In addition, insulin signaling is implicated in a negative feedback loop controlling sNPF expression and inhibiting food intake (124–127). In starved flies where insulin levels are low, sNPFR1 expression is upregulated resulting in facilitation of food search behavior (125). A microarray study revealed additional interaction partners of the sNPF system in *D. melanogaster*. The most pronounced and confirmed upregulated gene after sNPF administration is *mnb*, a Mnb/Dyrk1a kinase that activates the FOXO transcription factor through Sir2/Sirt1 deacetylase action (126). FOXO then in turn activates *snpf* transcription, providing a positive feedback loop. Mnb/Dyrk1a was localized in sNPFR1-expressing neurons, further evidencing an interaction with the sNPF signaling system (126). Genetic experiments using a combination of RNAi and overexpression lines of sNPFR1 and putative interaction partners revealed that activation of *mnb* transcription is attained through G_α*s*_, PKA and CREB (126). In addition, there is a positive feedback loop from CREB to sNPF in the regulation of energy homeostasis. CREB can dimerize with cAMP-regulated transcription coactivator (Crtc) to stimulate the expression of sNPF, resulting in an attenuation of the immune response and an increased starvation resistance. When this CREB/Crtc-dependent activation of sNPF is absent, the immune response is stimulated while depleting energy reserves (128).

MicroRNA (miRNA) regulation was also reported to be involved in the sNPF/Dilp signaling pathway in *D. melanogaster* IPCs. The conserved miRNA*miR-9a* is able to bind to the 3′-UTR of *sNPFR-1* mRNA and downregulates translation leading to a decreased production of sNPFR-1 in the IPCs (129). This inhibition of the sNPF signaling system results in a growth reduction. The NPF and sNPF system in *D. melanogaster* thus modulate feeding behavior in distinct manners but are both clearly essential for proper control of food intake and metabolism. The importance of the influence of both these systems on this delicate balance was demonstrated in a study investigating resistance to amino acid starvation. Reducing or increasing the expression of either *npf* or *snpf* drastically decreases the resistance to amino acid starvation and reduces lifespan on culture media deprived of amino acids (130).

### sNPF Signaling in Other Insects

Four peptides, termed “head peptides” were isolated from *A. aegyptii* head extracts using an FMRFamide-directed antiserum (131). These head peptides have long been assumed to be the sNPFs of *A. aegyptii*. However, they could not be detected in the completed *A. aegypti* genome by bioinformatics, nor in any tissue by mass spectrometric analyses. In contrast, the mature peptides encoded by the *snpf* gene of *A. aegypti* were found to be abundantly present in different parts of the CNS and also in the gut (132). Furthermore, *A. aegypti* NPYR1 is activated by sNPF-3, which indicates that this receptor is an sNPF receptor, and not an NPY receptor ortholog as stated in the study (133).

The intricate involvement of sNPF in feeding and metabolism has been demonstrated in many insects but depending on the species sNPF can act as a stimulating or inhibiting factor.

In *A. aegypti*, multiple sNPFs inhibit both serotonin-induced peristaltic contractions and ion transport of the anterior stomach using *in vitro* preparations, thus showing a negative modulation of serotonin-induced digestive action (59). *Aedes* sNPF receptor expression is significantly upregulated for 3 days post blood-feeding with a peak 48 h after the blood meal (133). In contrast to sNPFR expression, the amount of sNPF significantly drops in the antennal lobes following a blood meal. This drop in sNPF coincides with an inhibition of odor-mediated host-seeking behavior. Injection of sNPF is sufficient to mimic this inhibition in host-seeking behavior (134). In the mosquito *Culex quinquefasciatus*, sNPF precursor and receptor expression drops 27 h post sugar feeding, while a significant increase in sNPFR could be observed 27 h post blood-feeding (135). Thus, there seems to be a specific difference in the regulation of sNPF signaling according to the meal type. Taken together, these studies show that tissue-specific changes in components of the sNPF signaling pathway are instrumental in subtle modulation of feeding, food choice and food searching-related behaviors in mosquitoes.

In *S. gregaria*, sNPF transcription is inhibited in starved animals, while there is a temporal increase of sNPF transcription immediately after feeding (136). sNPF injection inhibits food intake and knockdown of sNPFR or sNPF precursor significantly increases food intake in S. gregaria (136, 137). These results prompted the idea that sNPF functions as a satiety factor that inhibits food intake in *S. gregaria*. Further characterization of the sNPF signaling pathway in *S. gregaria* revealed that the nutrient content in the haemolymph regulates sNPF transcription through the insulin signaling pathway (138).

In the cockroach *Periplaneta americana*, an upregulation of sNPF-IR cells in the midgut was shown upon starvation, while expression of digestive enzymes was drastically downregulated. In addition, refeeding significantly decreased sNPF IR within 3 h, suggesting an inhibitory function of sNPF on digestion (139). However, the possibility that the sNPF antiserum also recognizes NPF cannot be dismissed. *Ex vivo* incubation experiments of *P. americana* showed that sNPF directly inhibits the release of proteases, amylases, and lipases when co-incubated with midguts actively producing these digestive enzymes (139). sNPF injection in fed cockroaches increases locomotion to a level comparable to that of starved animals (140). This suggests that sNPF modulates locomotion in starved animals and that increasing circulating sNPF levels in fed animals override satiety and evoke food searching behavior.

In the silkworm *B. mori*, starvation caused decreased transcriptional levels of sNPFR, which again links sNPF to a crucial role in fed animals. In addition, mass spectrometric analysis revealed that sNPF levels in the brain decrease during starvation and increase upon refeeding (141). Injection of Bommo-sNPF-2 reduced the latency to feed (142). All these observations suggest that sNPF positively regulates food searching and feeding in this insect species.

A differential peptidomics study in the Colorado potato beetle, *Leptinotarsa decemlineata*, reveals that sNPF is absent in diapausing adults, but present in active beetles (143). This might indicate that sNPF is important in actively feeding animals having increased metabolic activity, while unnecessary in diapausing animals that are metabolically inactive.

In the honey bee, *A. mellifera*, food deprivation causes a significant upregulation of sNPF receptor transcription, pointing to a role of sNPF in starvation-resistance or the stimulation of foraging (68, 125). In another hymenopteran species, *S. invicta*, a downregulation of sNPFR transcription was observed in mated queens that were starved, compared to well-fed congeners (31), again suggesting the importance of sNPF signaling during feeding or metabolically active states.

Although sNPF has several other described functions, it seems to be mainly involved in the regulation of feeding.

### sNPF Signaling in Other Arthropods

The first crustacean sNPF was discovered in the giant freshwater prawn *Macrobrachium rosenbergii two* decades ago (144). A recent *in silico* study in this shrimp revealed the presence in the eyestalk and CNS of sNPF transcripts encoding four sNPF peptides (145). Another transcriptome study in ice krill *Euphausia crystallorophias* lead to the discovery of two sNPF precursors that cleave into several active peptides (146). A unique crustacean sNPF, containing an Asp residue in position 2, was found in *Daphnia pulex*. This makes Dappu-sNPF more similar to insect sNPFs than other crustacean sNPFs (147). Bioinformatic analysis of genomes and transcriptomes revealed the presence of sNPF and sNPF receptors in chelicerates (69). So far, no functional studies on crustacean or chelicerate sNPFs have been performed.

### sNPF Signaling in Nematodes

*C. elegans* sNPF neuropeptides display more sequence variation compared to other invertebrate sNPFs, which may be attributed to the extensive expansion and diversification of the sNPF signaling system in nematodes. Figure 4 shows that peptides derived from three distinct *C. elegans* sNPF neuropeptide precursors, FLP 15, FLP-18 and FLP-21, display the canonical motif XLRFa in accordance with the XXR(F/Y/W)amide C-terminal of sNPF in other protostomes.

A large scale phylogenetic analysis on bilaterian neuropeptide receptors indicated that also sNPF receptors underwent a large expansion in nematodes (1). Phylogenetic analysis showed that several *C. elegans* NPRs cluster with the *Drosophila* sNPF receptor, with NPR-6 being the closest (76). Also, other *C. elegans* receptors, including NPR-1,2,3,4,5, NPR-10, and NPR-13 cluster with the *Drosophila* sNPF receptor in agreement with the large expansion of sNPF receptors as postulated by Mirabeau and Joly (1). FLP-21 derived sNPFs have been shown to activate two candidate sNPF receptors, NPR-1, and NPR-2 in cell-based receptor assays (148–150). Similarly, FLP-18 derived peptides have been identified as a ligand for the NPR-1, NPR-4, and NPR-5 candidate sNPF receptors. FLP-15 has been shown to interact with the NPR-3 sNPF receptor (148, 149, 151, 152).

The role of nematode sNPF receptors in feeding has been examined for NPR-1, NPR-4, NPR-5, and their ligands. Although above-mentioned candidate sNPF receptors have been found in genomes of other nematodes as well, all the feeding-related functional data regarding sNPF in nematodes have been almost exclusively obtained in *C. elegans* (76, 80).

The sNPF receptor NPR-1 is a suppressor of food-dependent aggregation behavior in *C. elegans* (78). When food is present, some wild-type *C. elegans* strains, including the standard laboratory strain N2 Bristol, slow down their movement and disperse as solitary animals across a bacterial lawn. Other strains move faster and aggregate at the border of the food lawn (78). The difference between solitary and aggregation behavior comes down to a single amino acid change in NPR-1. Solitary worms have a gain-of-function allele of the neuropeptide receptor NPR-1, NPR-1 215V (valine at position 215), whereas aggregating animals have the natural isoform NPR-1 215F (phenylalanine at position 215). Worms with disturbed *npr-1* expression display solitary behavior (78). Bacterial odor influences the aggregation of *npr-1* animals, as does population density, although not when food is absent (153). Aggregation behavior is mainly driven by ambient oxygen levels. *C. elegans* escapes atmospheric levels of 21% oxygen, which signals exposure at the surface, by aggregating in groups of animals at the border of a bacterial lawn, where local oxygen levels are reduced.

The functions of NPR-1 in food-dependent aggregation behavior are mediated by two short NPF encoding genes, *flp-18* and *flp-21* (149). Deletion of *flp-21* increases the food-dependent aggregation behavior in NPR-1 215V and 215F worms, yet not to the level of the *npr-1* null mutant (149).

Mutants of *flp-18* have an altered metabolism, higher fat accumulation in the intestine and reduced oxygen consumption. Both *npr-4* and *npr-5* mutants display the same phenotypes suggesting that *flp-18*-mediated fat accumulation is executed by both receptors (151). Another effect of FLP-18 was observed in local search behavior. When wild type worms are removed from their food source, they increase their turning and reversing movements and explore the local area. When this withdrawal from food continues for a longer period, they start to search for food in bigger areas by inhibiting their turning and reversing behavior. Animals lacking *flp-18* fail to make this behavioral switch (151). This switch is regulated by AIY interneurons via NPR-4 signaling (154–156). AIY release of FLP-18 is also involved in dauer formation, a transition state aiding in survival when food is scarce. This effect of FLP-18 is controlled by NPR-5 in ASJ neurons (151).

Deletion of *npr-2* and *npr-7* have been associated with an increase in the intestinal fat storage, but the underlying mechanism seems different from the one observed in *npr-4* and *npr-5* mutants, since FLP-18, the ligand acting upon these last two receptors, is not active on NPR-2 and NPR-7 (151). The ligand of NPR-2 has been identified as FLP-21. NPR-2, along with NPR-1, seems to increase the adaptation to noxious stimuli in the absence of food. However, neither FLP-21 or FLP-18 are involved in this process (150). Although the same peptide is involved in the regulation of aggregation behavior, the action of FLP-21 upon NPR-2 in the modulation of fat storage has never been assessed.

### sNPF Signaling in Molluscs

A recent study in the Pacific oyster *Crassostrea gigas* pointed toward the homology of *C. gigas* LFRFamide neuropeptides and arthropod sNPFs (157). Yet, it is important to note that LFRFamide peptides lack the Arg residue at the fourth position from the C-terminus and have a Phe instead of a Leu residue at the third position from the C-termunus, which typified all known sNPFs at that time (158). Using *in silico* techniques, (157) found a Cragi-sNPFR-like receptor and showed that three Cragi-LFRFamide peptides from the same precursor activate this sNPF receptor in a dose-dependent manner (157). They further evidenced that the receptor is differentially expressed in males and females and is upregulated in starved oysters. These results may suggest a role in energy metabolism and reproduction (157) and make the system a convincing functional sNPF ortholog.

Even though the Cragi-sNPF-like system is the first one that was functionally characterized in molluscs, it is not the first LFRFamide (or sNPF) neuropeptide that was discovered in this phylum. Initially, LFRFamides or sNPFs were discovered in gastropods (159) and since then also in cephalopods and oysters (160, 161). In *A. californica*, LFRFamide peptides or sNPFs have an inhibitory effect in buccal neurons (162). In *L. stagnalis*, the sNPF-encoding gene is upregulated in response to *Trichobilharzia ocellata* infection, indicating a role in energy metabolism and reproduction (163). Another example is the involvement of sNPF peptides in feeding along with learning and memory in the cuttlefish *Sepia officinalis* (164, 165). For a detailed review see Bigot et al. (157) and Zatylny-Gaudin and Favrel (161).

### sNPF Signaling in Annelids

A bioinformatic study on *C. telata* predicts the presence of an LFRWamide neuropeptide encoding gene reported to be closely related to the mollusk gene (109) that encodes sNPF receptor activating LFRFamides or sNPFs (157). The genome of *C. telata* also encodes an sNPF receptor for which the activating ligand is currently unknown (114). In *Platynereis* the RYamide gene (110), encoding LFRWamides and XXRYamides, displays high sequence similarities with the LFRFamide (sNPF) precursor of mollusks (Figure 4). The *Platynereis* NKY receptor appears to be a candidate sNPF receptor. It was found to be activated *in vitro* (although at a high EC50 of 120 nM) by KAFWQPMMGGPLPVETRLASFGSRIEPDRTEPGSGPNGIKAMRYamide (111). This neuropeptide does, however, not belong to the NPF, nor the sNPF family. *In vivo* studies will be needed to demonstrate the cognate ligand of the annelid sNPF receptor. Knowledge on the function of sNPF signaling in annelids remains so far elusive.

### sNPF in Echinoderms

Information of sNPF in echinoderms is almost non-existing. Only in *A. rubens*, a GPCR that resembles an sNPF-type receptor rather than an NPF receptor has recently been identified (166).

## Conclusions

Despite their early evolutionary origin and subsequent evolutionary separation, NPF and sNPF neuropeptidergic signaling systems both control similar feeding aspects. In the species investigated, both of them converge to up- or downregulation of insulin signaling depending on the internal feeding state of the animal. It is remarkable to conclude that almost all invertebrate phyla retained both systems, even if their function in feeding is similar. It is, however, clear from the protostomian species investigated that both systems are needed for optimal regulation of feeding. This may suggest that both systems are probably controlling slightly different pathways underlying feeding behaviors. In vertebrates, sNPF signaling seems to have been lost during evolution, or may have evolved into the prolactin releasing peptide signaling system, which also regulates feeding and has been suggested to be orthologous to sNPF. Vertebrate long NPFs such as NPY, PPY and PP neuropeptide genes show an evolutionary expansion, either to compensate for the possible loss of sNPF, or to adapt to vertebrate-specific life styles and feeding.

## Author Contributions

LS and SZ conceptually designed the study. MF and IH equally contributed in writing the first draft of the manuscript. LS, SZ, LT, and IB wrote sections of the manuscript and critically revised it. All authors contributed to the final draft, have read and approved the submitted version.

### Conflict of Interest Statement

The authors declare that the research was conducted in the absence of any commercial or financial relationships that could be construed as a potential conflict of interest.
