# Supplementary material for: Regulation of Feeding and Metabolism by Neuropeptide F and Short Neuropeptide F in Invertebrates
Source: Front Endocrinol (Lausanne). 2019 Feb 19;10:64. doi: 10.3389/fendo.2019.00064 (PMC6389622; doi:10.3389/fendo.2019.00064)
Supplement: Supplementary file 1 [file Table_1.docx]

Supplementary Material

**Regulation of feeding and metabolism by Neuropeptide F and short Neuropeptide F in invertebrates**

Melissa Fadda*, Ilayda Hasakiogullari*, Liesbet Temmerman, Isabel Beets, Sven Zels and Liliane Schoofs

Department of Biology, Functional Genomics and Proteomics, KU Leuven, Naamsestraat 59, 3000 Leuven, Belgium

* These authors contributed equally to this work.

Corresponding author:

e-mail: liliane.schoofs@kuleuven.be

# Supplementary Table 1

List of NPF peptide sequences identified in nematodes (grey), platyhelminths (pink), molluscs (green), annelids (yellow), arthropods (blue), echinoderms (orange), cephalochordates and hemichordates (purple). The first and third columns represent species and according references of NPFs that have been biochemically isolated, or for which the transcript has been cloned. The second column represents representative amino acid sequences of the NPF(Y) peptides. The last two columns represent species and according references for NPF orthologues that have been only predicted by bioinformatics or for which NPY/PPY/PP/YY immunoreactivity has been reported. C-terminal amidation of peptides is denoted by a C-terminal small “a”. The carboxyterminal motif is indicated in bold face. For nematodes, the name of the peptides predicted to be NPF orthologues are given in brackets.

| **Species for isolated peptides** | **Peptide sequence** | **Reference** | **Species for predicted peptides** | **Reference** |
| --- | --- | --- | --- | --- |
| *C. elegans*  (*flp-34*) | ADISTFASAINNAG**RLRYa** | (Husson et al., 2014) | *C. briggsae, C. remanei, C. japonica, C. brenneri, B. malayi, W. bancrofti, D. immitis, O. ochengi, O. volvulus, L. loa, A. suum, A. caninum, N. brasiliensis, H. contortus, B. xylophilus, G. pallida, M. hapla, M. incognita* | (Li and Kim, 2014) |
|  | ALNRDSLVASLNNAE**RLRFa** |  |  |  |
| *C. elegans*  (*flp-27*) | EASAFGDIIGELKGKGLGG**RMRFa** | (Clynen et al., 2009) | *C. elegans, C. briggsae, C. remanei, C. japonica, C. brenneri, A. caninum, M. chitwoodi, M. hapla, M. incognita, M. javanica, M. paranaensis, N. americanus, R. similis, B. xylophilus, H. glycines, G. pallida* | (Li and Kim, 2010)  (Peymen et al., 2014)  ((Li and Kim, 2014)  (McCoy et al., 2014)  (McVeigh et al., 2005)  (Husson et al., 2007b) |
| *M. expansa* | PDQDSIVNPSDLVLDNKAALRDYLRQINEYFAIIG**RPRFa** | (Maule et al., 1991) | *P. littoralis, A. unipunctata, P. schultzei, P. hamatus, S. leucops, M. lineare, F. hepatica, H. diminuta, D. merlangi, M. corti, P. pollanicola, P. exiguous, P. indica* | (Reuter et al., 1995a)  (Reuter et al., 1995b)  (Magee et al., 1989)  (Fairweather et al., 1988)  (Maule et al., 1992)  (Hrckova et al., 1993)  (Gustafsson et al., 1995)  (Brennan and Ramasamy, 1996) |
| *A. triangulatus* | KVVHLRPRSSFSSEDEYQIYLRNVSKYIQLYG**RPRFa** | (Curry et al., 1992) |  |  |
| *S. japonicum,*  *S. mansoni* | AQALAKLMTLFYTSDAFNKYMENLDAYYMLRG**RPRFa** | (Humphries et al., 2004) |  |  |
| *L. stagnalis* | TEAMLTPPERPEEFKNPNELRKYLKALNEYYAIVG**RPRFa** | (Tensen et al., 1998) | *I. paradoxus, L. gigantea, C. gigas* | (Nuss et al., 2010)  (Veenstra, 2010)  (Bigot et al., 2014)  (Stewart et al., 2014) |
| *H. aspersa* | STQMLSPPERPREFRHPNELRQYLKELNEYYAIMG**RTRFa** | (Leung et al., 1992) |  |  |
| *A. californica* | DNSEMLAPPPRPEEFTSAQQLRQYLAALNEYYSIMG**RPRFa** | (Rajpara et al., 1992) |  |  |
| *L. vulgaris* | YAIVA**RPRFa** | (Smart et al., 1992) |  |  |
| *R. philippinarum* | LPDAMLSPPDRPNEFRSPGQLRSYLKALNDYYAIVG**RPRFa** | (Wang et al., 2017) |  |  |
| *C. gigas* | ALNDYYAIVG**RPRFa** | (Stewart et al., 2014) |  |  |
| *L. vannamei, M. marginatus* | KPDPSQLANMAEALKYLQELDKYYSQVS**RPRFa** | (Christie et al., 2011) | Present in all genomes and many neurotranscriptomes that have been analysed for NPF, except in *Triboleum castaneum* |  |
|  | KPDPSQLANMAEALKYLQELDKYYSQVSRPSPRSAPGPASQIQALENTLKFLQLQELGKLYSLRA**RPRFa** |  |  |  |
| *D. melanogaster* | SNSRPPRKNDVNTMADAYKFLQDLDTYYGDRA**RVRFa** | (Brown et al., 1999) |  |  |
| *N. vitripennis* | EPEPMARPTRPKVFESPEELRQYLDLVKEYYSLSG**KARYa** | (Hauser et al., 2010) |  |  |
| *A. mellifera* | EPEPMARPTRPEIFTSPEELRRYIDHVSDYYLLSG**KARYa** | (Hummon et al., 2006) |  |  |
| *R. prolixus* | VAAG**RPRFa**  NNRSPQ  LRLRF  NNRSPQ  LRLRF | (Ons et al., 2009) |  |  |
|  | NNRSPQL**RLRFa** |  |  |  |
| *D. pulex* | DGGDVMSGGEGGEMTAMADAIKYLQGLDKVYGQAA**RPRFa** | (Dircksen et al., 2011) |  |  |
| *A. pisum* | HPVTSTEVESIARPTRPKTFGSPDELRSYLDQLGQYLAVVS**RPRFa** | (Huybrechts et al., 2010) |  |  |
| *L. migratoria* | YSQVA**RPRFa** | (Clynen et al., 2009) |  |  |
| *S. gregaria* | YSQVA**RPRFa** | (Clynen et al., 2009) |  |  |
| *H. abietis* | pQELDNLYSPRS**RPRFa** | (Pandit et al., 2018) |  |  |
|  | QELDNLYSPRS**RPRFa** |  |  |  |
|  | MKLDQLYSSIA**RPRFa** |  |  |  |
| *H. zea* | QAA**RPRFa** | (Huang et al., 1998) |  |  |
|  | AA**RPRFa** |  |  |  |
| *A. aegypti* | RPQDDPTSVAEAIRLLQELETKH  AQHA**RPRFa** | (Stanek et al., 2002) |  |  |
|  | PTSVAEAIRLLQELETKHAQHA**RPRFa** |  |  |  |
|  | PEHFRNMDELNVYLDKLRQYYTILG**RPRFa** |  | *C. teleta* | (Nuss et al., 2010)  (Veenstra, 2011)  (Conzelmann et al., 2013) |
|  | DSSLDDIDVHPISVRSKAKPLPLPALFKNIDQLDKMLTDSFHSSSVYG**RPRFa** |  | *H. robusta* |  |
|  | AVEPPRRPEHFRNIEELNKYLAELRQYYTILG**RPRFa** |  | *A. pompejana*  (Peptides for *P. dumerilii, L. rubellus* and *Capitella. Sp* are not shown) |  |
|  | ATTGDKALDAILSGQYRH**HLRYa** |  | *A. filiformis* | (Martínez et al., 1993)  (Zandawala et al., 2017) |
|  | ALDAILSGQYRS**HLRYa** |  | *O. aranea* |  |
|  | ATTGDKALDAILSGQYRS**HLRYa** |  | *O. victoriae*  (Peptides for *A. rubens* and *M. glacialis* are not shown) |  |
|  | RVQRDASDYQATAPSRGASLAEWDRYLRELSLYRQYAD**IQRFa** |  | *S. kowalevskii* | (Mirabeau and Joly, 2013) |
|  | QEEEDVEAPEEGKYYKNLANYLRLLT**RQRYa** |  | *B. florida* |  |

# Supplementary Table 2

List of sNPF peptide sequences identified in nematodes (grey), molluscs (green), arthropods (blue) and annelids (yellow). The first and third columns represent species and according references of sNPFs that have been biochemically isolated, or for which the transcript has been cloned. The second column represents representative amino acid sequences of the sNPF peptides. C-terminal amidation of peptides is denoted by a C-terminal small “a”. The carboxyterminal motif is indicated in bold face. The last two columns represent species and according references for sNPF orthologues that have been predicted by bioinformatics or for which immunoreactivity has been reported. For nematodes, the names of the peptide precursors predicted to be sNPF orthologues are given in brackets. In *L. migratoria* and *S. gregaria*, X is Leu or Ile.

| **Species for isolated peptides** | **Peptide sequence** | **Reference** | **Species for predicted peptides** | **Reference** |
| --- | --- | --- | --- | --- |
| *C. elegans, C. briggsae*  (*flp-18*) | ^a^SVPG**VLRFa** | (Marks et al., 2001)^a^  (Husson et al., 2005)^b^  (Husson et al., 2009)^c^  (Husson et al., 2007a)^d^ | *C. remanei, C. japonica, C. brenneri, D. viviparus, A. caninum, M. javanica, A. ceylanicum, O. ostertagi, O. circumcincta, G. rostochiensis, P. Pacificus, H. contortus, S. stercoralis, M. chitwood, T.spiralis, M. hapla, M. incognita, T. muris, W. bancrofti,D. immitis, O. ochengi, O. volvulus, L. loa, A.suum, A. caninum, N. brasiliensis, H. contortus, S. ratti, B. xylophilus, Meloidogyne hapla, M. incognita, O. dentatum, P. redivivus, D. siriddicola* | (Lowery et al., 2000)  (McVeigh et al., 2005)  (Li and Kim, 2014) |
|  | ^b^EIPG**VLRFa** |  |  |  |
|  | ^b^SEVPG**VLRFa** |  |  |  |
|  | ^b^DFDGAMPG**VLRFa** |  |  |  |
|  | ^b^GAMPG**VLRFa** |  |  |  |
|  | ^b^SYFDEKKSVPG**VLRFa** |  |  |  |
|  | ^c^DVPG**VLRFa** |  |  |  |
|  | ^c^AYFDEKKSVPG**VLRFa** |  |  |  |
|  | ^d^EMPG**VLRFa** |  |  |  |
| *A. suum*  (*flp-18*) | AVPG**VLRFa** | (Cowden and Stretton, 1995) |  |  |
|  | GDVPG**VLRFa** |  |  |  |
|  | GFGDEMSMPG**VLRFa** |  |  |  |
|  | FGDEMSMPG**VLRFa** |  |  |  |
| *G. pallida*  (*flp-18*) | DEFVAPG**VLRFa** | (Kimber et al., 2001) |  |  |
|  | MPG**VLRFa** |  |  |  |
|  | AVPG**VLRFa** |  |  |  |
|  | AEVPG**VLRFa** |  |  |  |
|  | MPQ**VLRFa** |  |  |  |
| *A. suum*  (*flp-21*) | GLGPR**PLRFa** | (Cowden and Stretton, 1995) | *C. elegans, C. briggsae, C. remanei, C. japonica, C. brenneri, A. ceylanicum, B. malayi, H. contortus, M. hapla, N. americanus, O. ostertagi, P. penetrans, P. pacificus, R. similis, S. stercoralis, T. circumcincta, O. volvulus, O. ochengi, L. loa, D. immitis, W. bancrofti, O. circumcincta, N. brasiliensis, H. contortus, A. caninum, B. xylophilus, S. ratti, R. similis, G. pallida, M. incognita* |  |
| *C. elegans, C. briggsae*  (*flp-15*) | GGPQG**PLRFa** | (Husson et al., 2009)  (Husson et al., 2007a)  (Husson et al., 2006) | *C. remanei, C. japonica, C. brenneri, A. suum, O. circumcincta, N. brasiliensis, H. contortus, A. ceylanicum, T. circumcincta, N. americanus, O. ostertagi* | (McVeigh et al., 2005)  (Lowery et al., 2000))  (Li and Kim, 2014) |
|  | RGPSG**PLRFa** |  |  |  |
| *S. officinalis* | GN**LFRFa** | (Zatylny-Gaudin et al., 2010) | *P. fucata, A. irradians, I. paradoxus, H. diversicolor, A. californica, L. gigantea, S. officinalis, Idiosepius sp, Argopecten sp* | (Stewart et al., 2014)  (Zhang et al., 2012)  (Conzelmann et al., 2013) |
| *L. stagnalis* | GGS**LFRFa** | (Hoek et al., 2005) |  |  |
|  | T**LFRFa** |  |  |  |
| *A. californica* | ^a^GS**LFRFa** | ^a^(Cropper et al., 1994)  ^b^(Vilim et al., 2010) |  |  |
|  | ^a^ST**LFRFa** |  |  |  |
|  | ^a^GGA**LFRFa** |  |  |  |
|  | ^b^GAGT**LFRFa** |  |  |  |
|  | ^b^T**LFRFa** |  |  |  |
| *F. ferrugineus* | GS**LFRFa** | (Kuroki et al., 1993) |  |  |
|  | SS**LFRFa** |  |  |  |
| *C. gigas* | GS**LFRFa** | (Bigot et al., 2014) |  |  |
|  | SS**LFRFa** |  |  |  |
|  | GA**LFRFa** |  |  |  |
| *D. melanogaster* | SPSL**RLRFa** | (Baggerman et al., 2002)  (Lee et al., 2004) | Present in all genomes and many neurotranscriptomes that have been analysed for sNPF |  |
|  | ASRSPSL**RLRFa** |  |  |  |
|  | PQ**RLRWa** |  |  |  |
|  | PM**RLRWa** |  |  |  |
| *A. aegyptii* | KAVRSPSL**RLRFa** | (Predel et al., 2010) |  |  |
|  | SPSL**RLRFa** |  |  |  |
|  | APQL**RLRFa** |  |  |  |
|  | APSQ**RLRWa** |  |  |  |
| *P. americana* | ANRSPSL**RLRFa** | (Veenstra and Lambrou, 1995) |  |  |
| *H. abietis* | AGRSPQL**RLRFa** | (Pandit et al., 2018) |  |  |
| *A. pisum* | NQRSPSL**RLRFa** | (Huybrechts et al., 2010) |  |  |
| *D. pulex* | SDRSPSL**RLRFa** | (Dircksen et al., 2011) |  |  |
| *I. scapularis* | GGRSPSL**RLRFa** | (Neupert et al., 2009) |  |  |
|  | SPSL**RLRFa** |  |  |  |
| *A. mellifera* | GGRSPSL**RLRFa** | (Hummon et al., 2006) |  |  |
|  | SPSL**RLRFa** |  |  |  |
| *G. morsitans* | AQRSPSL**RLRFa** | (Caers et al., 2015) |  |  |
|  | SPSL**RLRFa** |  |  |  |
| *D. radicum* | SPSL**RLRFa** | (Zoephel et al., 2012) |  |  |
|  | AQRSPSL**RLRFa** |  |  |  |
| *N. vitripennis* | SGRSPSL**RLRFa** | (Hauser et al., 2010) |  |  |
|  | SPSL**RLRFa** |  |  |  |
| *L. migratoria* | SNRSPSX**RXRFa** | (Clynen et al., 2009) |  |  |
|  | SPSX**RXRFa** |  |  |  |
| *S. gregaria* | SNRSPSX**RXRFa** | (Clynen et al., 2009) |  |  |
|  | SPSX**RXRFa** |  |  |  |
| *L. decemlineata* | ARGPQL**RLRFa** | (Spittaels et al., 1996) |  |  |
|  | APSL**RLRFa** |  |  |  |
|  | SSPKPM**RLRWa** |  | *C. telata* | (Veenstra, 2011)  (Conzelmann et al., 2013) |
|  | **LFRWa** |  | *Platynereis sp.*  (Peptides for *P. dumerilii, Alvinella sp.* and *Pectinaria sp.* are not shown) |  |
